# Health systems strengthening through policy-level integration of community health worker programs into national health systems for improved health outcomes - scorecard metrics validation: A bifactor structural equation model approach

**DOI:** 10.3389/fpubh.2022.907451

**Published:** 2022-12-14

**Authors:** Lucia Mungapeyi Mupara, John Jules O. Mogaka, William R. Brieger, Joyce M. Tsoka-Gwegweni

**Affiliations:** ^1^Department of Public Health Medicine, University of KwaZulu Natal, Durban, KwaZulu-Natal, South Africa; ^2^Bloomberg School of Public Health, Johns Hopkins University, Baltimore, MA, United States; ^3^Faculty of Health Sciences, University of the Free State, Bloemfontein, South Africa

**Keywords:** community health work, organization & administration, delivery of health care, integrated, standards (integration metrics), structural equation modeling, quality of health care (improved health outcomes)

## Abstract

**Background:**

Subsequent to the demonstrated potential of community health workers (CHWs) in strengthening health systems to improve health outcomes, recent literature has defined context and guidelines for integrating CHW programs into mainstream health systems. However, quantitative measures for assessing the extent of CHW program integration into national health systems need to be developed. The purpose of this study was to validate a newly developed scale, Community Health Worker Program Integration Scorecard Metrics (CHWP-ISM), for assessing the degree of integration of CHW programs into national health systems in Sub-Saharan Africa (SSA).

**Methods:**

Data obtained through a pilot study involving a purposively selected sample of 41 participants selected from populations involved in CHW programs work in selected countries of SSA formed the basis of a 31-item bifactor model. Data were collected between June and December 2019. By applying a latent variable approach implemented with structural equation modeling, data analysis was mainly done using the R statistical environment, applying factor analysis procedures.

**Results:**

Dimensionality, construct validity, and the CHWP-ISM scale's internal consistency were assessed. Confirmatory factor analysis of the CHW-ISM bifactor model supported a co-occurring CHW integration general factor and six unique domain-specific factors. Both the comparative fit index (CFI) and Tucker–Lewis Index (TLI) fit indices were above 0.9, while the root mean square of the residuals (RMSR) was 0.02. Cronbach's alpha (α), Guttman 6 (Lambda 6), and Omega total (ω_t_) were above 0.8, indicating good scale reliability.

**Conclusion:**

Statistical significance of the bifactor model suggests that CHW integration has to be examined using factors that reflect a single common underlying integration construct, as well as factors that reflect unique variances for the identified six subject-specific domains. The validated CHWP-ISM could be useful to inform policy advisers, health systems, donors, non-governmental organizations, and other CHW program stakeholders with guidance on how to quantitatively assess the integration status of different components of CHW programs into respective critical functions of the health system. Improved integration could increase CHW program functionality, which could in turn strengthen the healthcare systems to improve health outcomes in the region.

## Introduction

Research evidence demonstrating the potential of community health workers (CHWs) in strengthening health systems to improve health outcomes has been growing (1–7), particularly their efficacy in improving the reach, impact, and efficiency of health services (8). In addition to improving health outcomes in general, evidence has also demonstrated their prospect in improving child health outcomes, particularly reducing childhood morbidity and mortality. (1, 9–12). This has been attributed to many reasons, including their placement as a bridge between health facilities and communities (13–16), which accord CHWs with an immense and unique advantage of proximity and availability in communities, to mobilize community members to identify and address their own health needs (13, 17).

However, this evident efficacy of CHWs in delivering community-based preventive and curative services is being truncated by varying, subjective, or at worst lack of CHW program integration into national health systems (11, 18–20). In an attempt to review practical strategies to reduce the Under 5 Mortality Rate (U5MR), the 2015 Renewed Promise to Child Survival underscored the need to strengthen health systems to deliver high-quality high-impact interventions (HIIs) (21) for child health. Scott et al. highlighted that the integration of CHWs with health systems necessitates their inclusion into public policies that direct national service delivery, human resources for health, health financing, medical products and technologies, health information and leadership, and governance critical functions of the health system (22). The call for this health workforce cadre to be integrated into national health systems has been stressed (8, 22–29) and in particular, in Sub-Saharan Africa (SSA) region (4, 8).

Recent literature has defined the context, mechanisms, and guidelines for integrating CHW programs into mainstream health systems (1, 19, 25–27, 30–36). However, much of what is currently in use for assessing CHW program integration is based on qualitative measures. Although qualitative metrics add valuable information, given that some integration determinants cannot be captured by quantitative measures, such metrics can be subjective. Qualitative metrics may include human experience or judgment as a factor in measurement and information that can often be difficult to measure due to ambiguity. On the other hand, there are advantages to measuring CHW integration quantitatively because the extent can often be clearly expressed as a ratio, percentage, or even average that can be compared across two or more program settings. Basing CHW integration metrics on quantitatively validated models increases transparency and consistency. To date, little research has been done to assess CHW programs' integration quantitatively or the use of quantitative integration metrics to assess CHW program integration into national health systems, at both policy and implementation levels.

This study validated newly developed scorecard metrics for measuring the extent of CHW programs' integration into national health systems (CHWP-ISM) in Sub-Saharan Africa (38) at the policy level. The paper tests the proposed CHW integration metrics scale's construct validity, dimensionality, and reliability. The items, generated and reported in Mupara et al. (38), included some suggestions proposed by Boateng et al. (37) as best practices for developing and validating scales for health.

## Methodology

### Theoretical considerations, item selection, and study conceptual framework

**Figure 1:**
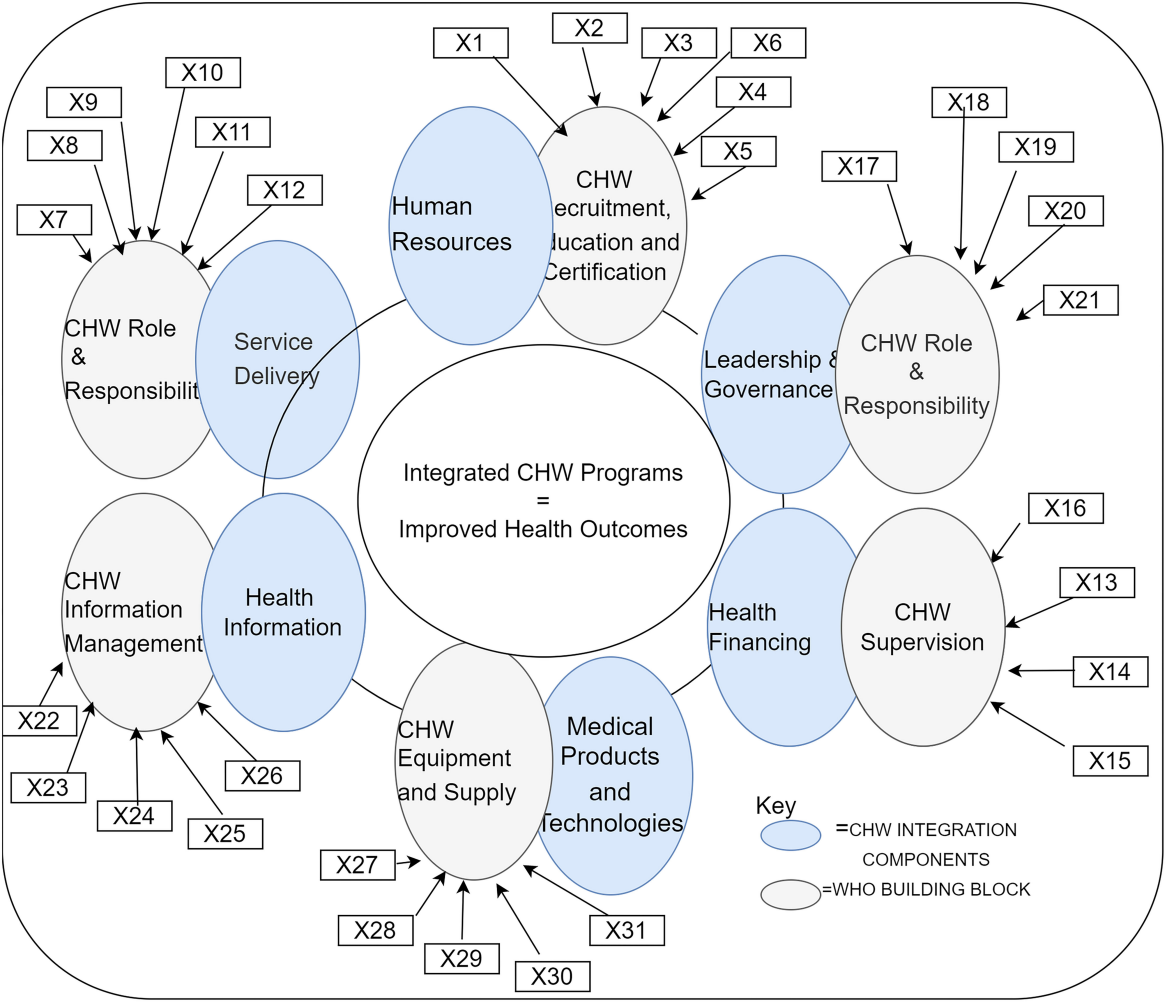
Study conceptual framework (X1–X31 as indicated in Table 1). Source: Adapted from the study by Mupara and Mogaka (38).

The present study was based on the earlier reported study (38), which is part of a wider study undertaken to generate items suitable for assessing the extent of integration of CHW programs into national HS. The study (Mupara et al. (38) study on integration indicators) was based on WHO's building blocks for strengthening health systems (HSS) (Figure 1). In this study, CHW program integration at the policy level has been defined as the policy-level guidelines and directives that specify how CHW programs should be mainstreamed into national HS. These policy guidelines were viewed in line with the WHO Health systems (HS) building blocks, namely, service delivery, human resources for health, health information, medical products and technologies, health financing, and leadership, and governance (39). Therefore, for the purpose of this study, six matching integration domains whose indicators could be measured to ascertain the extent of CHW program integration, specifically at the policy level, were identified. These were CHW Recruitment, Education, and Certification (REC) (23, 25, 26); CHW Role and Responsibilities (R&R); CHW Remuneration; CHW Supervision; CHW Information Management; and CHW Equipment and Supplies (25, 26, 32, 33, 40). Whereas, the earlier study had identified a hundred (100) items for measuring the extent of CHW integration into national health systems, this study took a subset of 31 items, with CHW REC and CHW R&R having six indicators each. The rest of the parameters had five indicators each except for CHW Remuneration which had four indicators. Item selection and distribution for each integration variable are indicated in Table 1 and Figure 1.

**Table 1:** The six WHO critical functions and community health worker (CHW) integration program components, integration parameters, and integration indicators (measures).

**Who building block**	**CHW integration components**	**Integration parameters/ indicators**	**Code**
Human resources for health	1. CHW Recruitment, Education and Certification	1.1 Government policy stipulates selection criteria for CHW recruitment.	X1
		1.2 Government policy provides guidance on selection process followed when recruiting CHWs.	X2
		1.3 Government sponsors CHW training	X3
		1.4 Policy documents indicate how CHW Training Programs are offered by accredited (institutional and training providers using accredited curricula	X4
		1.5 Policy documents describe CHW Training modalities including duration, scope, mode of delivery etc.	X5
		1.6 Policy documents provide guidelines on end of course competency-based evaluation and certification	X6
Service delivery	2. CHW Roles and Responsibilities	2.1 Government policy stipulates scope of services delivered by CHWs	X7
		2.2 Policy documents indicate the need for role agreement among CHW, community and health system	X8
		2.3 Policy documents describe the linkage between CHW cadres and the formal health system	X9
		2.4 Policy documents clarify the interventions and services delivered by CHWs to community and health system	X10
		2.5 Policy documents acknowledge CHWs as the first tier/ point of contact between community and health system	X11
		2.6 Policy documents describe the mechanisms of counter-referral of patients between CHWs and health facilities	X12
Health financing	3. CHW Remuneration	3.1 Government policy indicate the need for CHW to sign formal contractual agreements stipulating CHW working conditions, job responsibilities, and rights.	X13
		3.2 Government fully funds CHW incentives	X14
		3.3 Policy documents describe standardized package of financial Incentives for CHWs	X15
		3.4 Policy documents describe standardized package of non-financial Incentives for CHWs	X16
Governance and leadership	4. CHW Supervision	4.1 Policy documents describe how the government takes part in CHW supervision through the health system structures	X17
		4.2 Policy documents defines how community health work should be managed across all levels of the health system	X18
		4.3 Policy documents provides guidelines on appropriate supervisor training and supervisor-to-supervisee ratio	X19
		4.4 Policy documents suggest CHW Supportive supervision Strategies	X20
		4.5 Policy documents define how CHW Performance evaluation should be conducted by supervisors	X21
Health information	5. CHW Information management	5.1 Data confidentiality and security articulated in government policy documents	X22
		5.2 Policy documents stress the need for community and facility data consolidation	X23
		5.3 Government policy articulates how CHW generated data should be integrated into the national health information and management system	X24
		5.4 Policy documents define CHW role in data collection	X25
		5.5 Policy documents define CHW supervisor role in data collection	X26
Medical products and technologies	6. CHW supplies and equipment	6.1 CHW equipment and supplies are integrated into the procurement and supply processes of the national supply chain plan as defined by policy documents	X27
		6.2 Policy documents provide a list of selected medicines, commodities, and supplies administered by CHWs in line with national essential medicines list for respective countries	X28
		6.3 Policy documents provide guidance on protocols for CHWs to access emergency stock to facilitate smooth stock out management and reduce disruptions in community programs.	X29
		6.4 Policy provides guidance on how community health providers should restock-commodities, medicines, and equipment.	X30
		6.5 Policy provides guidance on safe disposal of medical waste generated through CHW service	X31

### Study participants

**Figure 2:**
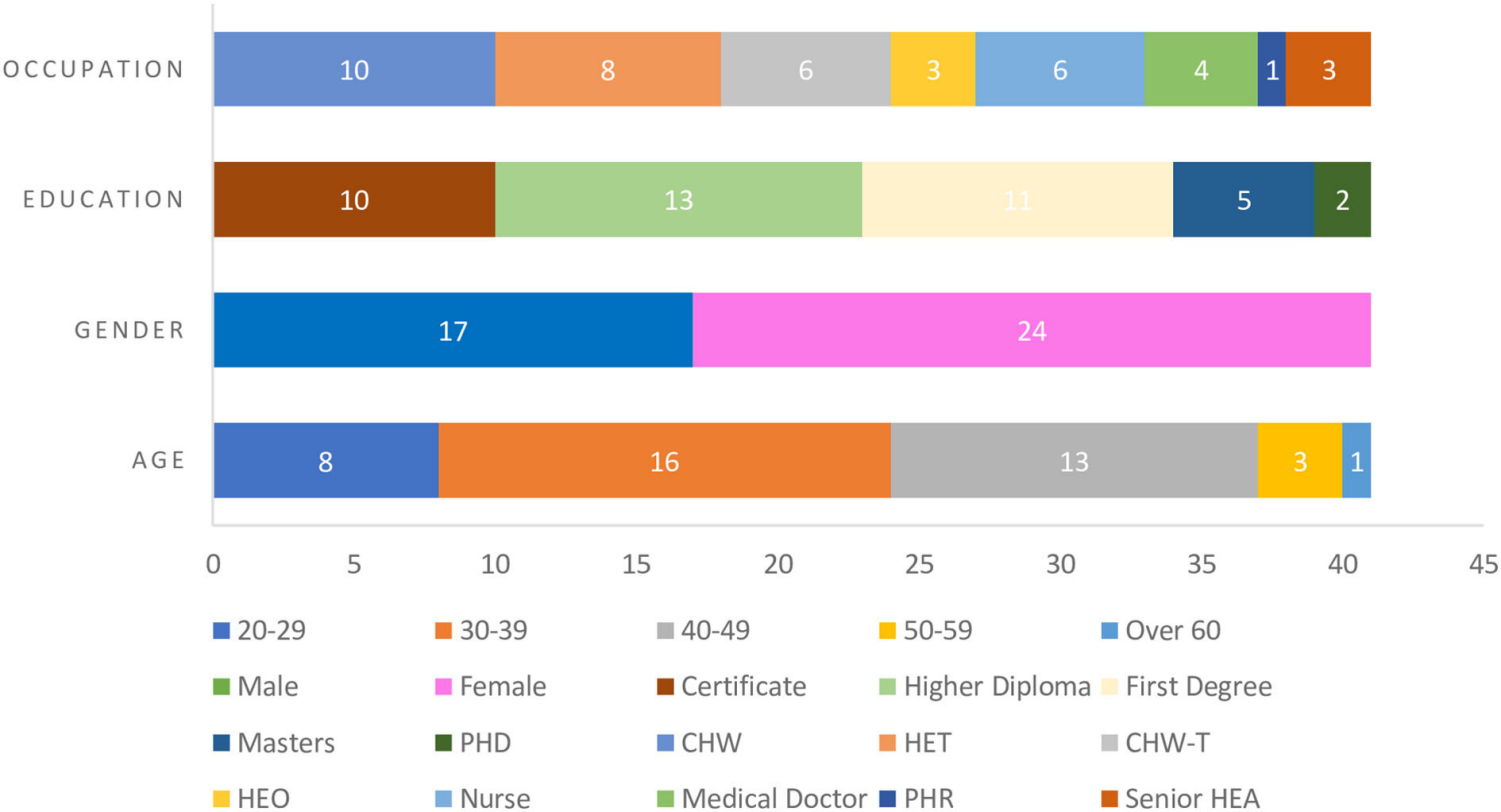
Demographic characteristics of study participants. CHW, community health worker; HET, Health Education Technician; CHW-T, CHW trainer; HEO, Health Education Officer; PHR, Public Health Researcher; HEA, Health Education Assistant.

Purposive sampling was employed to identify and invite a sample of 45 healthcare providers who were involved with CHW program work at different levels of service delivery in SSA. Out of the invited 45 healthcare providers, 41 agreed to participate in this study. Figure 2 gives a graphical presentation of the composition of the study participants, comprising of CHWs, senior CHWs, CHW trainers/lecturers, health education technicians and officers, nurses, medical doctors, and public health researchers. Their educational qualifications ranged from certificate, diploma, degree, master's, and Ph.D. holders. In selecting the sample, the study particularly focused on the diversity of participants in terms of educational levels and different healthcare cadres involved in community health work at different levels and settings to ensure data source triangulation.

### Procedure

This study was guided by the general principles of the Nuremberg Code, the Declaration of Helsinki, and the Institutional Review Board. A study package was delivered to selected eligible participants by hand, courier services or email depending on the geographical location (distance) of potential participants between January and April 2019. The questionnaires were self-administered. The package included an invitation letter with a study description, a consent form, and a questionnaire. The first section of the questionnaire was designed to elicit demographic information about participants, including their age, gender, educational level, and occupation. The participants were asked to rate their considered opinion on a 5-point (“strongly agree” to “strongly disagree”) Likert-type scale in order to express how certain items could be used to measure the extent of integration of CHW programs into national health systems. The items that participants responded to are listed in Table 1. Forty-one participants successfully completed the survey. Completed questionnaires were retrieved back from participants through hand delivery and couriered services. Data were captured in Excel, cleaned, and processed for analysis using R. There were no missing data.

### Statistical considerations

The CHWP-ISM bifactor model was assessed using a latent variable approach implemented with structural equation modeling. The study constructed latent variables (constructs) that were hypothesized to have varying influences on CHW integration. Their relationship with corresponding integration (observable) indicators was then statistically tested using the bifactor modeling method (41, 42) through exploratory and confirmatory factor analyses (EFA/CFA) (43).

The 31 items were grouped to reflect specific domains, which are as seen in the study by Tellegen and Waller (44). The specific domains for this study were CHW REC; CHW R&R; CHW Remuneration; CHW Supervision; CHW Information Management, and CHW Supplies and Equipment as shown in Table 1. A bifactor latent structure analysis, as explained in the study by Reise et al. (45), was done to assess the scale's unidimensional-multidimensional nature. Bifactor models allow for the assessment of hierarchical models of constructs, examining whether indicators contribute to specific (unique) factors over and above their contribution to a general factor (42). This modeling approach enabled us to derive (*via* EFA) and test (*via* CFA) the most optimal way to present the CHWP-ISM scale: either as a conceptually broad “CHW integration” unidimensional factor, or as a multidimensional scale made of domain-specific factors; or as a blend of the two, reflecting both general and sub-domain factors. The single factor scale reflected the variance common among all observed measures in the checklist, while the multidimensional checklist reflected additional common variance among item clusters, corresponding to content-specific subdomain constructs. First, a factor analysis model was specified using the “omega” (46) function in “psych” (47) version 1.8.12 R package, i.e., an exploratory factor analysis (EFA). The “omega” function incorporates Leiman transformations (41) in estimating bifactor structures. Next, a CFA model was specified using “lavaan” version 0.6–3 (48, 49) in R version 3.6.1 (50), which provided the fitted item to use with the “omegaFromsem” function for CFA.

Criteria for item retention for the final model were based on factor loading >0.20 on either the general factor or any of the specific factors. The goodness of fit was tested using global and local fit indices. Given that this was a new scale development, cutoff points were according to the following fit criteria: SRMR≤ 0.1, TLI and CFI ≥0.80 for acceptable fit, root mean square error of approximation (RMSEA)≤ 0.06, and SRMR≤ 0.08 (51). Besides, both modification indices and item content theory were used in model modification. Coefficient alpha (α) and 3 omega coefficients (ω_T_, ω_h_, and ω_hs_) from the ‘omegaSem' output were used to examine the internal consistency and reliability of the CHWP-ISM scale.

## Results

### Demographic characteristics of participants

There were 41 participants out of the initial 45 invited (88% response rate). There were more female participants than male participants (51%). Those with a diploma or above as the highest academic qualifications were the majority (75%), while 24 % of the participants who hold certificate qualifications are CHWs. All participants who took part in the study had a tertiary education qualification and were all proficient in the English language. The rest of the participants' demographic profiles varied greatly and are summarized in Figure 2.

### Correlations and descriptive statistics

Descriptive statistics of the data were obtained, including an analysis of outliers, which was defined as any value that is greater than three standard deviations above or below the mean. Skew statistics were assessed using the “mardia” function in “psych” version 1.8.12 (52) R package that applies Mardia's tests for multivariate skewness and kurtosis. Skew values ranged from (–) 1.87 to (–) 0.85 and kurtosis (*k*) statistics for 30 items ranged from −0.49 to 1.91, with the 31st item indicated at *k* = 3.61. However, this outlier did not alter results and so it was included in the dataset. The data have a general normal distribution. This justified the use of the maximum likelihood estimation method (ML) in carrying out further analysis using the data.

### Variable correlation

**Figure 3:**
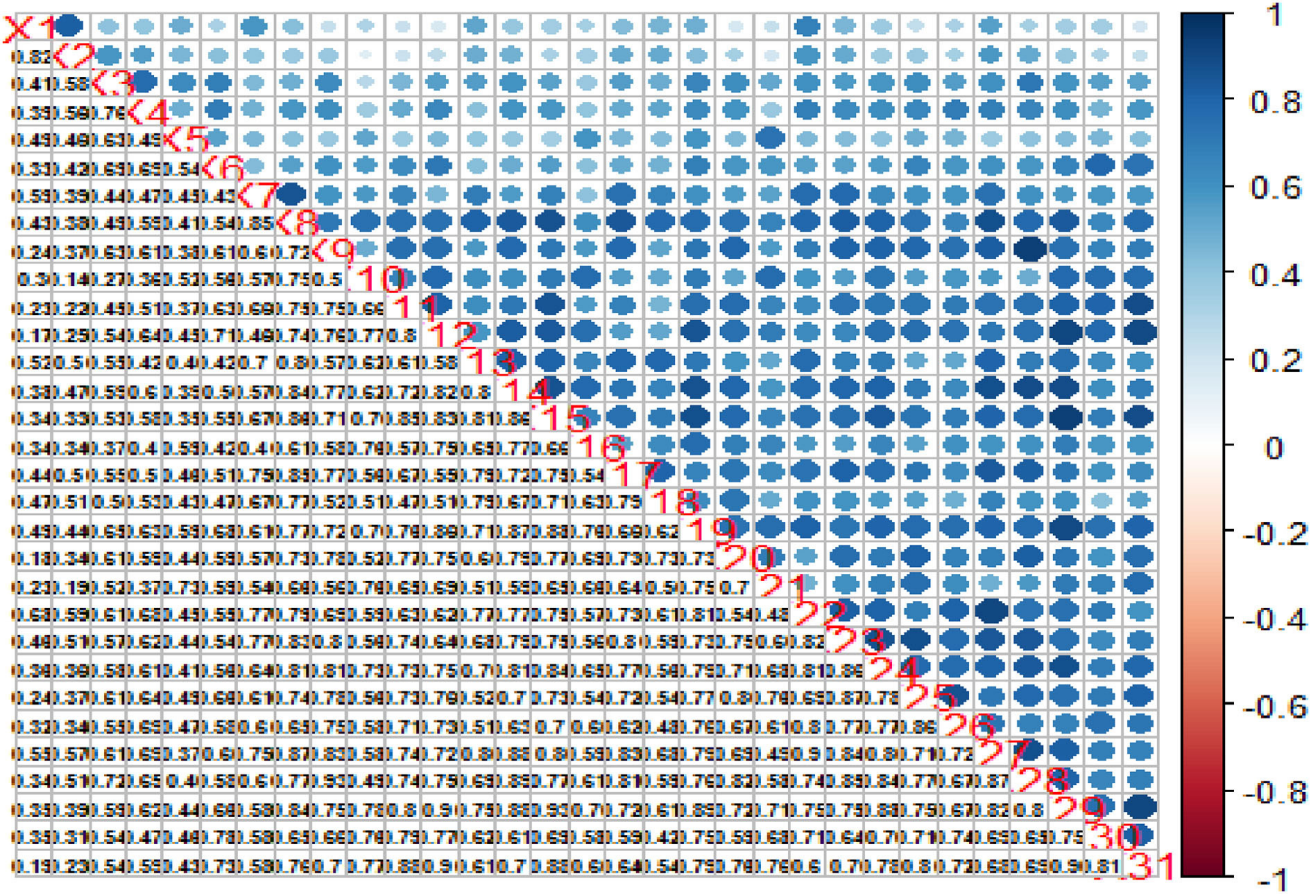
Variable correlation matrix.

We used the R package “corrplot” version 0.84 (53) to visualize the data correlation matrix. The correlogram in Figure 3 displays variables in the correlation matrix and how they relate with each other. In the upper triangle, positive correlations are displayed in blue and negative correlations in red color. Color intensity and the size of the circle are proportional to the correlation coefficients, helping to identify “groups” of variables that share a strong relationship with each other (hierarchical clustering). The lower triangular correlation matrix displays the actual correlation values.

### Construct validity

**Figure 4:**
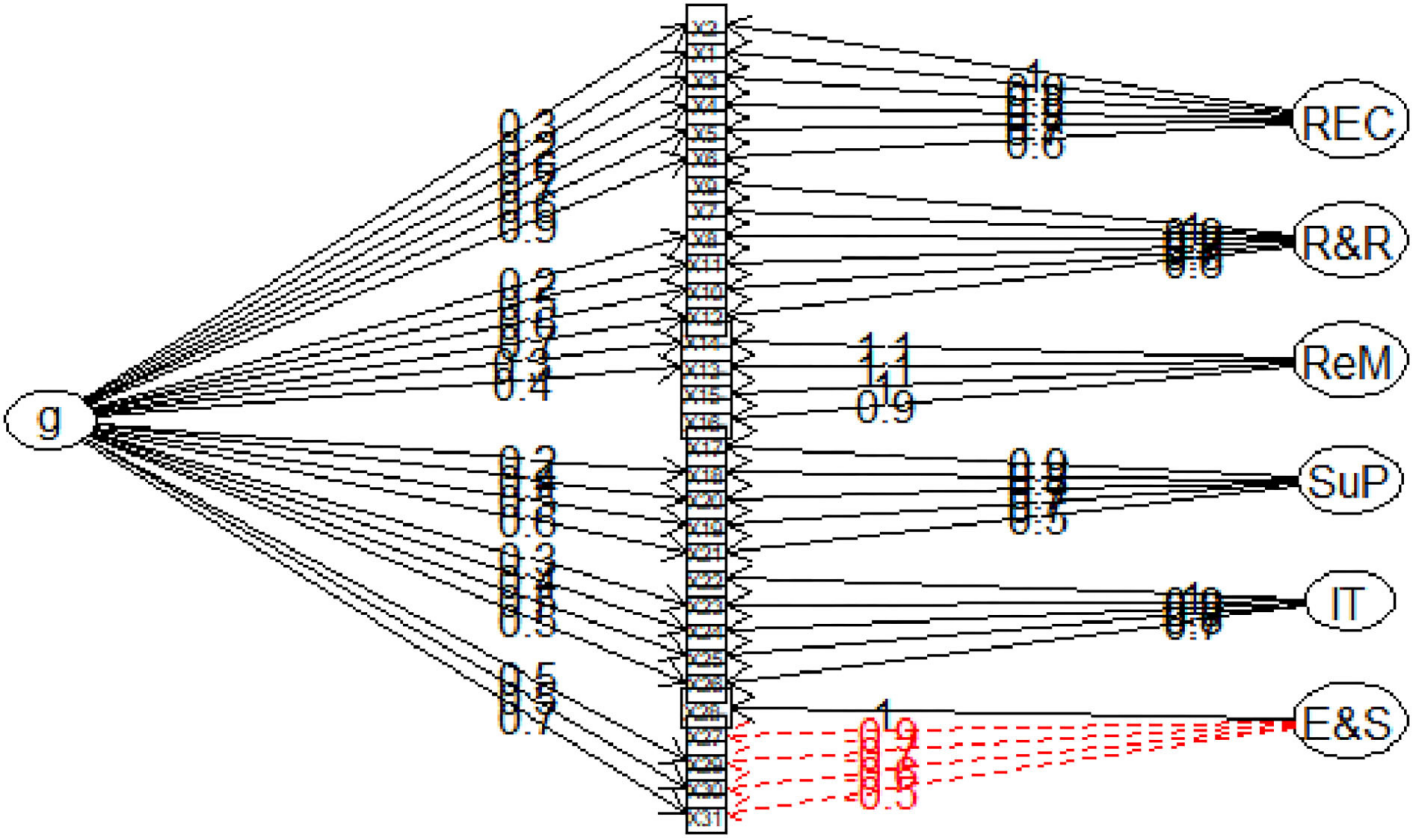
Exploratory bifactor model for CHW-ISM scale. g, the conceptualized general factor indicative of communalities of all items; REC, “CHW Recruitment, Education and Certification” construct; R&R, “CHW Roles and Responsibilities” construct. ReM, “CHW Remuneration”; SuP, “CHW Supervision”; IT, “CHW Information management”; E&S, “CHW supplies and equipment” Items labels (x1–x31) correspond to items as listed in Table 1.

We estimated the CHW-ISM bifactor model by finding the number of latent factors underlying the 31 integration items (observed variables) with an initial EFA. Figure 4 shows the item correlations between observed indicators and the latent variables. Table 2 shows the EFA solution that indicates seven eigenvalues, with five that are >1.0 and two closer to 1, implying seven extractable factors (one general factor and six content-specific factors). This is consistent with our prior hypothesis on CHW-ISM structure: a general (unidimensional) factor and six content-specific unique factors. A further examination of the EFA factor structure in Table 2 shows that, for the most part, the loading pattern of the bifactor solution has items more or less perfectly settling into respective domain-specific parcels that speak to the six domains as hypothesized (Table 1). More generally, however, the items have more loading on the general factor (CHW program integration). This implies a factor structure in which convergent and discriminant validity are evident by the high loadings to the specific factors, with most factors loading above 0.70. Loading on the general factor, however, greatly varies: some items' loadings are < 0.2 (not shown because we set cutoff level of ≥ 0.2 loadings), while others are as high as 0.9. This is strong evidence that the main CHW integration construct can be sub-scaled into its separate, distinct but correlated elements/subscales.

**Table 2:** Exploratory and confirmatory bifactor solutions.

**CHW integration model factor analysis**
Alpha: 0.98
G.6: 0.98
Omega Hierarchical: 0.82
Omega H asymptotic: 0.84
Omega Total 0.98
With eigenvalues of:
g F1* F2* F3* F4* F5* F6*
15.28 1.20 1.30 1.15 1.20 0.81 0.80
The root mean square of the residuals is 0.02
The df corrected root mean square of the residuals is 0.03
RMSEA index = 0.06 and the 90% confidence intervals are NA 0.09
Explained common variance of the general factor = 0.7
Total, general and subset omega for each subset
g F1* F2* F3* F4* F5* F6*
Omega total for total scores and subscales 0.98 0.88 0.90 0.88 0.81 0.82 0.84
Omega general for total scores and subscales 0.82 0.73 0.71 0.73 0.56 0.69 0.69
Omega group for total scores and subscales 0.03 0.15 0.19 0.15 0.26 0.12 0.15
The following analyses were done using the Lavaan package
Omega Hierarchical from a confirmatory model using sem = 0.42
Omega Total from a confirmatory model using sem = 0.99
With loadings greater than 0.2
g F1* F2* F3* F4* F5* F6* h2 u2 p2
X1 0.25 0.94 0.95 0.05 0.07
X2 0.29 0.96 1.01 −0.01 0.08
X3 0.59 0.80 0.99 0.01 0.35
X4 0.66 0.78 1.05 −0.05 0.41
X5 0.63 0.70 0.89 0.11 0.45
X6 0.89 0.61 1.16 −0.16 0.68
X7 0.91 0.83 0.17 0.00
X8 0.21 0.89 0.83 0.17 0.05
X9 0.97 0.95 0.05 0.00
X10 0.57 0.58 0.66 0.34 0.49
X11 0.48 0.66 0.66 0.34 0.35
X12 0.67 0.56 0.77 0.23 0.58
X13- 0.39 1.12 1.41 −0.41 0.11
X14- 0.28 1.13 1.35 −0.35 0.06
X15- 1.00 0.99 0.01 0.00
X16- 0.87 0.76 0.24 0.00
X17 0.91 0.86 0.14 0.04
X18 0.21 0.80 0.68 0.32 0.06
X19 0.51 0.74 0.81 0.19 0.32
X20 0.37 0.74 0.69 0.31 0.20
X21 0.62 0.54 0.67 0.33 0.57
X22 0.20 0.96 0.96 0.04 0.04
X23 0.32 0.88 0.87 0.13 0.12
X24 0.45 0.79 0.83 0.17 0.24
X25 0.50 0.76 0.82 0.18 0.30
X26 0.48 0.72 0.76 0.24 0.30
X27 −0.93 0.86 0.14 0.01
X28- 0.98 0.96 0.04 0.00
X29 0.52 −0.69 0.75 0.25 0.36
X30 0.48 −0.64 0.63 0.37 0.37
X31 0.71 −0.51 0.76 0.24 0.66
With eigenvalues of:
g F1* F2* F3* F4* F5* F6*
6.1 3.9 3.6 4.3 2.9 3.4 3.0
mean percent general = 0.24 with sd = 0.22 and cv of 0.92
Explained common variance of the general factor = 0.22
Total, general, and subset omega for each subset
g F1* F2* F3* F4* F5* F6*
Omega total for total scores and subscales 0.99 1.32 0.86 1.25 0.85 0.94 0.75
Omega general for total scores and subscales 0.42 0.43 0.14 0.03 0.17 0.17 0.38
Omega group for total scores and subscales 0.29 0.89 0.73 1.21 0.68 0.77 0.37
CFI 0.99
TLI 0.99

h2, item communalities; u2, item uniqueness; p2, item-explained common variance; G.6, Guttman's Lambda 6; RMSR, root mean square residual (recommended value≤0.06). Factors in the Table: “g”, F1, F2, F3, F4, F5, and F6 correspond to constructs “g”, REC, R&R, ReM, SuP, IT, and E&S in Figure 4.

Column “h2” in Table 2 presents values for item-explained common variance (i.e., percent communality) due to the general factor. These values suggest most items from all constructs equally form good candidates for inclusion in a unidimensional (one common factor) item set, i.e., they form the core of a unidimensional CHW-ISM scale. This is evidenced by their large communality values (h2).

To examine the extent the CHW-ISM bifactor model fits the data, we examined a number of absolute and relative fit indices (51). The root mean square of residual (RMSR), the square root of the difference between the residuals of the sample covariance matrix and the hypothesized covariance model, was indicated as 0.02 (acceptable fit is below 0.08 (51)). The comparative fit index (CFI) and Tucker Lewis Index (TLI) above.95 are currently considered an indicator of excellent fit (51, 54), and CHW-ISM Scale's CFI and TLI were above this threshold, indicating excellent fit. Average Variance Extracted (AVE) was indicated as 0.7, which is above the recommended > 0.5 for establishing convergent validity. The above factor loading and model fit statistics present evidence for the construct validity of the CHWP-ISM scale.

### CHWP-ISM scale reliability

To test the bifactor model's dimensionality, we examined whether item factor loading of the scale could be explained by one general factor, plus several specific factors corresponding to each of the scale's facets (dimensions).

As seen in Table 2, the standardized coefficient alpha was given as 0.98, implying that 98% of the observed score variance can be attributed to the “true score” variance. However, coefficient alpha has been said to be limited in explaining variance when the data are multidimensional, as in the case of a bifactor model (55). The factor structure in Table 2 suggests an essentially congeneric model and not a tau-equivalent model that the alpha coefficient is particularly suited for (56). Therefore, we also used omega coefficients (41, 45, 46) to indicate the construct reliability of the PMI measurement scale. It has been noted that omega coefficients provide a more accurate approximation of a scale's reliability (57).

Omega total (ω_t_) accounts for the variance due to the general factor, as well as the group factors. From Table 2, it is indicated that ω_t_ = 0.98. Even though ω_t_ is appropriate for varying factor loadings as seen in the bifactor model, its value is influenced by all modeled sources of common variance and includes item-specific variance as an error (58). To further clarify sources of variance, we used coefficient omega hierarchical (ω_h_) and coefficient omega hierarchical subscale/group (ω_hS_). Coefficient ω_h_ estimates the proportion of variance in total scores that can be attributed to a single general factor. From Table 2, when ω_t_ and ω_h_ are compared, we noted that 42% of all reliable variance in the total scores (0.42/0.98) can be attributed to the general factor, assumed to reflect individual differences in the trait of CHW integration. Fifty-seven percent (0.95–0.36) of the reliable variance in total scores is attributable to the multidimensionality caused by the four group factors. This implies that the CHW-ISM scale is principally (~40–60) unidimensional-multidimensional, reflecting a co-occurring CHW integration general factor and six unique content-specific factors. This is also supported by the explained common variance (ECV) due to the general factor, which is 0.22, implying that the general factor explains 22% of the common variance extracted, with 78% of the common variance spread across the six unique group factors.

On the other hand, the unique variance associated with each of the six subscales once the variance associated with the general (unidimensional) factor is partitioned out is indicated by coefficient ω_hs._ This index reflects the reliability of a subscale score after controlling for the variance due to the general factor (59). In this case, ω_hs_ was reported as REC = 0.89; R&R = 0.73; ReM = 1.21; Sup = 0.68; IT = 0.77; and E&S = 0.38. These values imply that the reliability of subscales is adequate and justifies the use of the CHW-ISM subscales for any future quantitative investigations of CHW integration into health systems.

## Discussion

With the growing importance of CHW programs on the global health agenda, comes the responsibility to create a scientific foundation for CHW integration metrics and evaluation. However, CHW integration into national health systems is a broad concept. This implies that the 'CHW integration' construct has a more diverse content, making it more reasonable to question whether it can be adequately measured as a single unidimensional construct or as a multidimensional construct composed of many sub-constructs. In this study, a bifactor model was applied to explore the unidimensional-multidimensional structure of the newly defined CHW integration measurement tool. Study findings provide evidence to the question as to whether the data set had a strong enough common factor (unidimensional), or had a more complex multidimensional (content-specific subcategory) structure. Furthermore, the tool's validity and reliability as a quantitative measure of CHW integration into healthcare systems were investigated and documented. This presented a strong basis for an objective means of measuring CHW integration into national health systems across healthcare jurisdictions.

Although factor loading was adequate, the bifactor CFA model did not suggest a perfect unidimensional CHW integration construct, as shown in Figure 4. Furthermore, strong content-specific correlations did not point to the main common CHW integration construct. Therefore, the combined CHW-ISM bifactor scale presents a better and more plausible model which can explain scale reliability at subscale (multidimensional) as well as at full-scale (unidimensional) levels. This is because the bifactor model shows how all items simultaneously measure both the common CHW integration trait and at the same time account for the variance of each item as influenced by domain-specific (subscale) groupings.

## Conclusion

This study aimed at validating the CHW Integration Metrics Scorecard (CHWP-ISM) for assessing the degree of integration of CHW programs into the national health system in SSA. We proposed that this validated CHWP-ISM can be used to evaluate the extent of integration of health interventions aimed at strengthening health systems through the WHO HS building blocks. The metrics scorecard can be used to pair component health interventions (integration variables) with corresponding WHO HS building blocks that they feed into, and then the extent of integration can then be determined considering the integration variables.

The process of integration stage is used to determine if there are country policies that speak to the inclusion of CHW program integration variables into respective health system building blocks. Evidence of integration zooms into the specific guidelines developed from the policies detailing the day-to-day running of the CHW programs in a way that exhibits that they are part and parcel of the health system. Having established that the above is in place, the presence or absence of integration indicators will be used to score all the aspects that make up the specific integration parameter. Thereafter, the aggregation of the integration indicators can be judged against the scale to determine the extent of integration.

In general, the CHWP-ISM can be used to review CHW programs' extent of integration at all levels of the health system be they local, district, provincial, or national. Particularly, to reconnoiter the interaction between the respective components of the health intervention under study and its corresponding WHO building block of the health system. It is hoped that the use of this metrics scorecard to assess the extent of integration of CHWs could better strengthen health systems to improve health outcomes in Sub-Saharan Africa.

One weakness of the study is the assumption that all participants understand CHW policy issues at the same level. Further studies should be conducted with participants with the same level of understanding of CHW policy.

## Data availability statement

The raw data supporting the conclusions of this article will be made available by the authors, without undue reservation.

## Ethics statement

The studies involving human participants were reviewed and approved by Biomedical Research Ethics Committee, University of KwaZulu-Natal. The patients/participants provided their written informed consent to participate in this study.

## Author contributions

All authors listed have made a substantial, direct, and intellectual contribution to the work and approved it for publication.
